# Effectiveness of GLP-1 Receptor Agonists in Patients With Polycystic Ovary Syndrome: A Systematic Review and Meta-Analysis of Randomised Controlled Trials

**DOI:** 10.7759/cureus.106751

**Published:** 2026-04-09

**Authors:** Sukainnya Buragohain, Indrani Sarma, Dibyajyoti Saikia, Himangshu Malakar, Bipul Kumar Das, Murchana Khound, Joonmoni Lahon

**Affiliations:** 1 Pharmacology, All India Institute of Medical Sciences, Guwahati, Guwahati, IND; 2 Obstetrics and Gynaecology, All India Institute of Medical Sciences, Guwahati, Guwahati, IND; 3 Paediatrics, All India Institute of Medical Sciences, Guwahati, Guwahati, IND

**Keywords:** exenatide, glp-1 receptor agonists, liraglutide, metformin, polycystic ovary syndrome, systematic review and meta analysis

## Abstract

Polycystic ovary syndrome (PCOS) is a complex endocrine-metabolic disorder, and while metformin is widely used in its management, its efficacy remains variable. Glucagon-like peptide-1 receptor agonists (GLP-1 RAs) have emerged as potential alternatives due to their metabolic benefits. In this systematic review, we have evaluated the effectiveness of GLP-1 RAs on body mass index (BMI), homeostatic model assessment of insulin resistance (HOMA-IR), and total testosterone (TT) in women with PCOS. Randomised controlled trials (RCTs) comparing the effect of GLP-1 RAs with metformin, standard, or placebo on BMI, HOMA-IR, and TT in women with PCOS were selected following Preferred Reporting Items for Systematic Reviews and Meta-Analysis (PRISMA) 2020 guidelines. A comprehensive literature search was conducted in various databases, including PubMed, Scopus, Embase, MEDLINE, Web of Science, Cochrane, and CINAHL through May 20, 2025. Eighteen RCTs comparing GLP-1 RAs with placebo, metformin, or standard therapy were included. Quality assessment was done using Cochrane's 'Risk of Bias tool (RoB2)'. Review Manager (RevMan) version 5.4.1 (The Cochrane Collaboration, London, United Kingdom) was used to perform random-effects meta-analysis. Continuous outcomes were pooled as mean differences (MD) when measured on the same scale and as standardised mean differences (SMD) when the scales differed, each with a 95% confidence interval (CI). Inter-study heterogeneity among the trials was assessed using the chi-squared test for heterogeneity, with I² to quantify the level of heterogeneity. The certainty of evidence was determined using the Grading of Recommendations Assessment, Development, and Evaluation (GRADE) approach. GLP-1 RAs significantly reduced BMI compared with control interventions (MD: −1.09 kg/m²; 95% CI: −1.80 to −0.38; p = 0.003), with liraglutide showing superiority over both metformin and placebo in subgroup analyses. Insulin resistance improved significantly (SMD: −0.38; 95% CI: −0.61 to −0.16; p = 0.001), particularly with exenatide. However, no significant overall effect on TT levels was observed (SMD: −0.10; 95% CI: −0.38 to 0.18; p = 0.49). Risk of bias was generally low, with minor concerns in select domains. Funnel plots suggested minimal publication bias. The certainty of evidence was moderate for BMI and HOMA-IR and very low for TT. GLP-1 receptor agonists are effective in improving metabolic outcomes in PCOS, particularly in reducing BMI and insulin resistance. However, their effect on androgen levels remains inconclusive. These agents may represent a promising therapeutic option, especially in overweight or obese women with PCOS, though further large-scale studies are needed to confirm long-term benefits.

## Introduction and background

Polycystic ovary syndrome (PCOS), also known as hyperandrogenic anovulation or Stein-Leventhal syndrome, is one of the most prevalent endocrine-metabolic disorders affecting women of reproductive age [1]. Characterised by heterogeneous clinical features including oligo- or anovulation, hyperandrogenism, and polycystic ovarian morphology, PCOS is frequently accompanied by metabolic dysfunctions such as obesity and insulin resistance [2]. These metabolic impairments significantly increase the long-term risk of type 2 diabetes, cardiovascular disease, and endometrial cancer in affected individuals [3]. Current therapeutic approaches primarily focus on symptom management, targeting menstrual irregularities, hyperandrogenic features, and metabolic disturbances. Metformin, a well-established insulin sensitiser, is widely recommended as a second-line agent for managing metabolic aspects of PCOS [4]. However, the therapeutic response to metformin, particularly with respect to weight reduction and insulin sensitivity, remains variable.

Glucagon-like peptide-1 receptor agonists (GLP-1 RAs), initially developed for the management of type 2 diabetes, have emerged as promising alternatives owing to their multifaceted metabolic benefits [5]. These agents enhance insulin secretion, suppress appetite, slow gastric emptying, and promote weight loss. However, their effects on androgen-related outcomes remain comparatively less certain and are supported by a smaller and more heterogeneous body of evidence. Preliminary studies suggest that GLP-1 RAs may be more effective than metformin in reducing body mass index (BMI), improving insulin resistance, and regulating menstrual cycles in women with PCOS [6]. Nevertheless, despite these promising findings, existing trials vary considerably in sample size, methodology, and outcome reporting, rendering it difficult to draw firm conclusions. Given the growing interest in GLP-1 RAs as a potential therapy for PCOS, this meta-analysis aims to synthesise the available randomised controlled trial (RCT) evidence to evaluate the comparative effectiveness and safety of GLP-1 RAs in patients with PCOS.

## Review

Methods

Search Strategy

We systematically searched PubMed, Scopus, Embase, Medical Literature Analysis and Retrieval System (MEDLINE), Web of Science, Cochrane, Cumulative Index to Nursing and Allied Health Literature (CINAHL) databases, and the Clinical Trials Registry India (CTRI) registry to identify RCTs comparing GLP1-RAs versus placebo, metformin, or standard treatment among women diagnosed with PCOS after registration in PROSPERO with reference ID CRD42024576690. The search utilised the following medical subject headings (MeSH) terms and their combinations: polycystic ovary syndrome, GLP-1 receptor agonist, exenatide, lixisenatide, liraglutide, semaglutide, dulaglutide, and tirzepatide. The search was restricted to RCTs conducted in humans.

Inclusion and Exclusion Criteria

Completed RCTs comparing GLP-1 RAs (monotherapy or in combination) with standard treatment, metformin, or placebo in separate study arms were included. Eligible studies must involve females diagnosed with PCOS using different diagnostic criteria, like the National Institutes of Health (NIH) criteria, the Rotterdam criteria, and the National Institute of Child Health and Human Development (NICHD) criteria [7], who are either overweight or obese, undergoing treatment, and receiving at least one GLP-1 RA. Studies were excluded from this meta-analysis if they did not meet the criteria of being RCTs involving patients with a confirmed diagnosis of PCOS based on established criteria. Non-randomised studies, observational studies, case reports, reviews, and conference abstracts without full-text availability were excluded. Trials that did not evaluate GLP-1 RAs (e.g., liraglutide, exenatide) as the primary intervention, or those lacking a placebo or standard treatment comparator, were also excluded. Additionally, studies that did not report relevant metabolic or reproductive outcomes (such as weight change, insulin resistance, or androgen levels), were published in languages other than English without an available translation, or lacked sufficient data for analysis, were not included.

Endpoint Analysis

Our primary outcomes of interest included BMI, homeostasis model assessment of insulin resistance (HOMA-IR), and total testosterone (TT). We performed data extraction and quality assessment for studies that met the inclusion criteria. For continuous outcomes, we pooled the mean difference (MD) when studies reported outcomes on a common scale and the standardised mean difference (SMD) when measurement scales differed, each with 95% confidence intervals (CI), using a random-effects model.

Quality Evaluation of Included Studies

We followed Preferred Reporting Items for Systematic Reviews and Meta-Analysis (PRISMA) 2020 guidelines [8]. The risk-of-bias tool for randomised trials, version 2 of the Cochrane (RoB 2) [9], was used independently by two authors to assess the risk of bias in the included studies, and disagreements were resolved through a consensus after discussing the reasons for the discrepancy. For individually randomised studies, we assessed RoB in five domains: (i) the randomisation process; (ii) deviations from intended interventions; (iii) missing outcome data; (iv) measurement of the outcome; and (v) selection of the reported result. Domain 1 was assessed at the study level, and the other domains were assessed at the result level. For each domain, we made a judgement of high RoB, low RoB, or some concerns. We used the signalling questions and algorithm and considered whether to override the algorithm result, recording our reasons and supporting evidence. The small-study effect was investigated using a funnel-plot analysis of point estimates based on study weights.

Statistical Analysis

Meta-analysis was performed using Review Manager (RevMan) version 5.4.1 (The Cochrane Collaboration, London, United Kingdom) [10]. For continuous outcomes, effect sizes were expressed as MD for outcomes measured on the same scale (e.g., BMI in kg/m²) and as SMD for outcomes pooled across studies with differing scales or variability structures, each with 95% CI. Heterogeneity was assessed using the chi-squared test and quantified with the I² statistic. A random-effects inverse-variance model was used for all pooled analyses, and a p-value <0.05 was considered statistically significant.

Results

Study Selection

**Figure 1:**
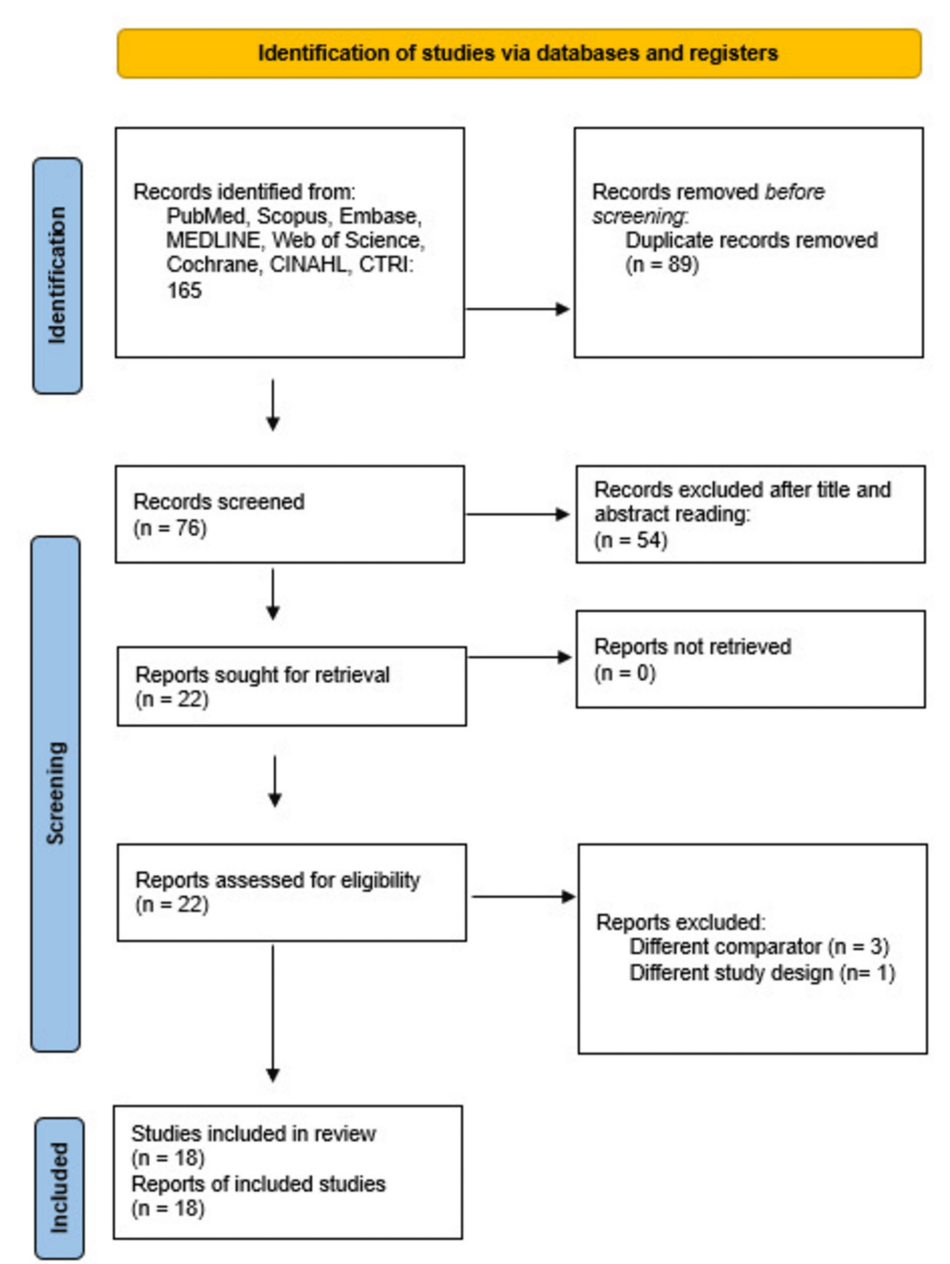
PRISMA 2020 flowchart depicting the selection process of the studies PRISMA: Preferred Reporting Items for Systematic Reviews and Meta-Analysis [8]

As illustrated in Figure 1, the initial search identified 165 records from databases, including PubMed, Scopus, Embase, MEDLINE, Web of Science, Cochrane, CINAHL, and CTRI. After removing 89 duplicate records, 76 records remained for screening. Following title and abstract screening, 54 records were excluded. A total of 22 reports were sought for retrieval, and all were successfully retrieved. These 22 reports were assessed for eligibility, of which four were excluded (three due to a different comparator and one due to a different study design). Finally, 18 studies met the eligibility criteria and were included in the systematic review. All included studies were published in peer-reviewed journals.

Study Characteristics

Baseline characteristics of the 18 RCTs included in the meta-analysis are depicted in Table 1. Overall, despite variability in sample size and specific inclusion criteria, the baseline profiles across studies were broadly comparable in terms of age, BMI, and metabolic characteristics, supporting the appropriateness of pooling these studies for meta-analysis while acknowledging underlying clinical heterogeneity.

**Table 1:** Baseline characteristics of the studies included in the meta-analysis PO: orally; s.c.: subcutaneous; mg: milligram; mcg: microgram; CRD: calorie-restricted diet; PHO phenotype: PCO morphology (P), hyperandrogenic features (H) and oligo-amenorrhea (O); phenotype A: hyperandrogenism + oligo-anovulation + polycystic ovaries; OW/OB: overweight/obese

Sl no.	Year	Author	Study population	Intervention	Sample size	Age (in years)	Control	Sample size	Age (in years)
1	2022	Karen E. Elkind-Hirsch, et al. [11]	nondiabetic, premenopausal, BMI ≥30 kg/m^2^	Liraglutide 3 mg once daily for 32 weeks	55	18–45	Placebo	27	18–45
					44 (analysed)			23 (analysed)	
2	2014	Mojca Jensterle Sever, et al. [12]	obese women with PCOS, more than 18 years old, premenopausal, obese (BMI ≥ 30 kg/m^2^)	Liraglutide 1.2 mg once daily s.c. for 12 weeks	11	31.5±6.4	Metformin 1000 mg twice daily for 12 weeks	14	31.3±9.4
3	2015	Mojca Jensterle, et al. [13]	PCOS, overweight/obese (BMI ≥25 kg/m^2^)	Liraglutide 1.2 mg once daily s.c. 12 weeks	15	18 years to menopause	Metformin 1000 mg twice daily PO for 12 weeks	15	18 years to menopause
					14 (analysed)			13 (analysed)	
4	2015	Mojca Jensterle, et al. [14]	obese women with newly diagnosed PCOS (aged 27.6±7.2 years, BMI 39.5±6.2 kg/m²)	Liraglutide 1.2 mg OD sc for 12 weeks	14 completed	29.5±7.7	Metformin 1000 mg twice daily PO for 12 weeks	14 completed	25.3±5.2
5	2017	Mojca Jensterle, et al. [15]	obese women with PCOS, type A phenotype, age 18 years to menopause and obesity (BMI ≥ 30 kg/m^2^)	Liraglutide 3 mg once daily for 12 weeks	15	34.6±6.1	Metformin 1000 mg twice daily and Liraglutide 1.2 mg once daily for 12 weeks	15	31.6±5.9
					14 completed			14 completed	
6	2021	Rui-Lin Ma, et al. [16]	PCOS, overweight/obese (BMI ≥25 kg/m^2^)	Metformin 500 mg three times daily + Exenatide once weekly 2 mg	25	30.10±4.52	Metformin 500 mg daily, increased to 1500 mg daily over 2 weeks	25	28.17±4.40
					19 (analysed)			21 (analysed)	
7	2017	Zheng S, et al. [17]	OW/OB patients with PCOS, 18-40 years, BMI: 24-28 kg/m^2^	Exenatide 10 mcg once daily for 1 week, followed by 10 mcg twice daily for 12 weeks	41	27.2±3.1	Metformin 500 mg twice daily followed by 1000 mg twice daily	41	27.7±2.7
					31 completed			32 completed	
8	2017	Xin Liu, et al. [18]	PCOS, overweight/obese (BMI ≥25 kg/m^2^)	Exenatide treatments (10 mcg once daily, then 10 mcg twice daily s.c.) 12 weeks	88	27.69±3.80	Metformin 500 mg once daily for 1 week, then 1000 mg twice daily for 12 weeks	80 (analysed)	27.93±2.70
					78 (analysed)				
9	2023	Zhang Y, et al. [19]	18-45 years, BMI ≥24 kg/m^2^, PCOS	Dulaglutide 1.5 mg weekly sc + CRD 6 months	35	30.31 (28.58–32.05)	CRD	33	28.64 (27.09–30.18)
10	2023	Jensterle M, et al. [20]	PCOS, Obese	Semaglutide 0.25 mg once weekly for 2 weeks	10	35 (mean)	Placebo	10	35 (mean)
				Semaglutide 0.5 mg once weekly for 2 weeks				9 (analysed)	
				Semaglutide 1 mg once weekly for 8 weeks					
11	2021	Jensterle M, et al. [21]	PCOS, obese, BMI 36.1 ± 3.9 kg/m^2^, 33.7 ± 5.3 years, Phenotype A	Semaglutide 0.5 mg once weekly for 4 weeks	15	33.7±5.3	Placebo	15	33.7±5.3
				Semaglutide 1.0 mg once weekly for 12 weeks	13 (analysed)			12 (analysed)	
12	2021	Tao T, et al. [22]	PCOS with prediabetes, 18 and 45 years, BMI ≥25 kg/m^2^	Exenatide 10 mcg/day for 4 weeks	61	18–45	Metformin 1500 mg 1 week increase to 2000 mg per week for 4 weeks	61	18–45
				Exenatide 20 mcg/day for 4-12 weeks	50 (analysed)			50 (analysed)	
13	2023	Gan J, et al. [23]	overweight or obese, BMI ≥ 25 kg/m^2^, aged 18–40 years	Exenatide 2 mg SC once a week + Metformin 500 mg thrice daily for 12 weeks	28	29.96±5.39	Metformin 500 mg thrice daily for 12 weeks	22	28.45±4.74
14	2022	Xing C, et al. [24]	PCOS phenotype B, 18-40 years, BMI ≥24 kg/m^2^, barrier contraception	Metformin 1000 mg twice daily PO + Liraglutide 1.2 mg once daily sc for 12 weeks	30	25.85±4.45	Met 1000 mg twice daily PO	30	23.52±4.65
					27 (analysed)			25 (analysed)	
15	2023	Mingyu Liao, et al. [25]	overweight PCOS women, BMI ≥ 24 kg/m^2^	Liraglutide, 1.2–1.8 mg/day + Metformin, 1500 mg/day for 12 weeks	35	18–50	CPA/EE (2 mg/day: 2 mg cyproterone acetate and 35 μg ethinylestradiol) + Metformin (1500 mg/day) for 12 weeks	35	18–50
					30 completed			30 completed	
16	2017	Malin Nylander, et al. [26]	age 18 years or above, PCOS according to Rotterdam criteria, BMI ≥ 25 kg/m^2 ^and/or insulin resistance	Liraglutide 1.8 mg/day for 6 months	48	31.4 (24.6–35.6)	Placebo	24	26.2 (24.8–31.5)
					44 (analysed)			21 (analysed)	
17	2008	Karen Elkind-Hirsch, et al. [27]	overweight oligoovulatory women with PCOS, 18–40 yr and overweight/obese (BMI > 27 kg/m^2^)	Exenatide 10 mcg twice daily for 24 weeks	20	28.2±1.1	Metformin 1000 mg twice daily	20	27.7±1.3
					14 completed			14 completed	
18	2018	Salamun V, et al. [28]	PCOS, obese, Infertile, classic PHO phenotype, BMI ≥30 kg/m^2^, age ≤38 years, non-diabetic	Metformin 1000 mg twice daily po + Liraglutide 1.2 mg once daily s.c. for 12 weeks	14	30.1±3.6	Met 1000 mg twice daily PO	14	31.1±4.7
					13 (analysed)				

Meta-Analysis

The primary outcomes of the study related to the effectiveness of GLP-1 RA included the following parameters of BMI, HOMA-IR, and TT.

**Figure 2:**
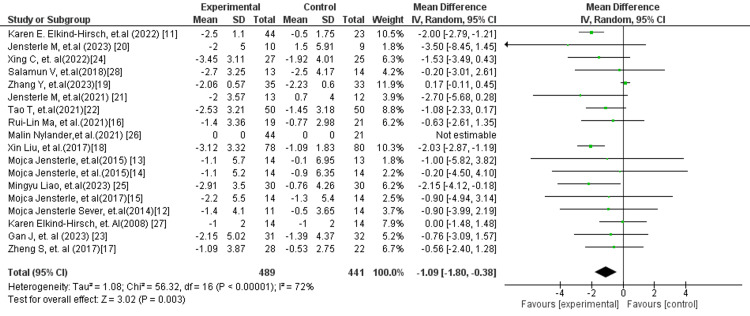
Forest plot for BMI overall

In Figure 2, the overall pooled analysis of all included studies demonstrated a statistically significant reduction in BMI in favour of GLP-1 RAs compared to control interventions (MD: −1.09 kg/m²; 95% CI: −1.80 to −0.38; p = 0.003). However, substantial heterogeneity was observed (I² = 72%), indicating variability across studies that may be attributable to differences in study populations, intervention types, or treatment durations. Despite this heterogeneity, the direction of effect consistently favoured GLP-1 RA therapy, supporting its beneficial role in weight reduction.

**Figure 3:**
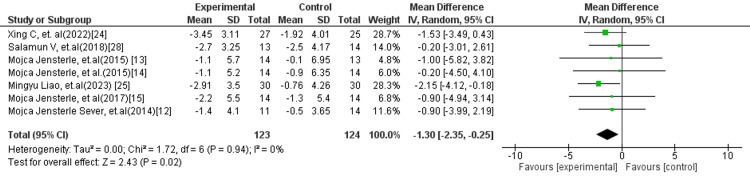
Forest plot comparing liraglutide versus metformin for BMI

**Figure 4:**
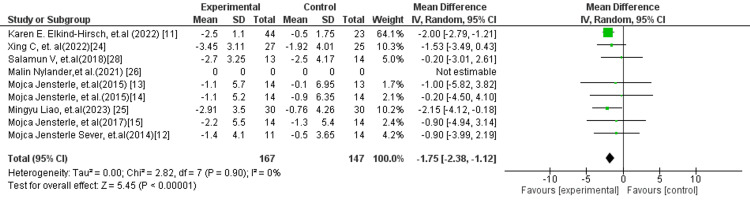
Forest plot comparing liraglutide versus placebo/standard treatment for BMI

In subgroup analyses, more consistent and robust findings were observed. The comparison of liraglutide versus metformin (Figure 3) demonstrated a statistically significant reduction in BMI favouring liraglutide (MD: −1.30 kg/m²; 95% CI: −2.35 to −0.25; p = 0.02), with no observed heterogeneity (I² = 0%), indicating a stable and reproducible effect across studies. Similarly, the liraglutide versus placebo/standard treatment subgroup (Figure 4) showed a highly significant reduction in BMI (MD: −1.75 kg/m²; 95% CI: −2.38 to −1.12; p < 0.00001), again with no heterogeneity (I² = 0%), reinforcing the reliability of liraglutide’s effect in weight reduction.

**Figure 5:**
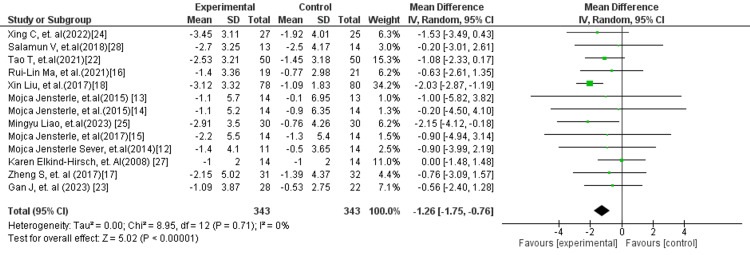
Forest plot comparing GLP-1 RA versus standard treatment for BMI GLP-1 RA: Glucagon-like peptide-1 receptor agonist

**Figure 6:**
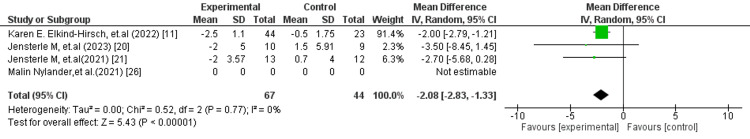
Forest plot comparing GLP-1 RA versus placebo for BMI GLP-1 RA: Glucagon-like peptide-1 receptor agonist

The pooled analysis of GLP-1 receptor agonists versus standard treatment (Figure 5) also demonstrated a significant decrease in BMI (MD: −1.26 kg/m²; 95% CI: −1.75 to −0.76; p < 0.00001; I² = 0%), suggesting a class effect of GLP-1 RAs over conventional therapies. Furthermore, the comparison of GLP-1 receptor agonists versus placebo (Figure 6) revealed the largest magnitude of BMI reduction (MD: −2.08 kg/m²; 95% CI: −2.83 to −1.33; p < 0.00001; I² = 0%), indicating strong efficacy in reducing body weight when compared to placebo controls.

**Figure 7:**
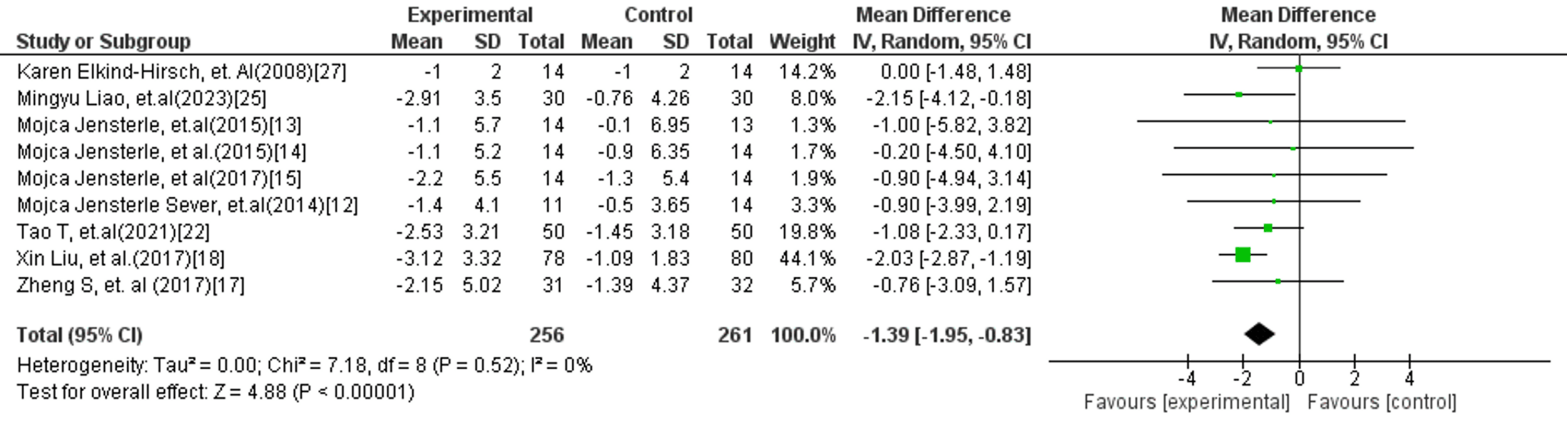
Forest plot comparing GLP-1 RA versus GLP-1 RA combined with metformin for BMI GLP-1 RA: Glucagon-like peptide-1 receptor agonist

Another subgroup meta-analysis (Figure 7) of nine studies comprising 517 participants demonstrated that GLP-1 RA monotherapy resulted in a significantly greater reduction in BMI compared to GLP-1 combined with metformin (MD = −1.39; 95% CI: −1.95 to −0.83; p < 0.00001). No heterogeneity was observed among the included studies (I² = 0%), indicating a high level of consistency in the findings.

**Figure 8:**
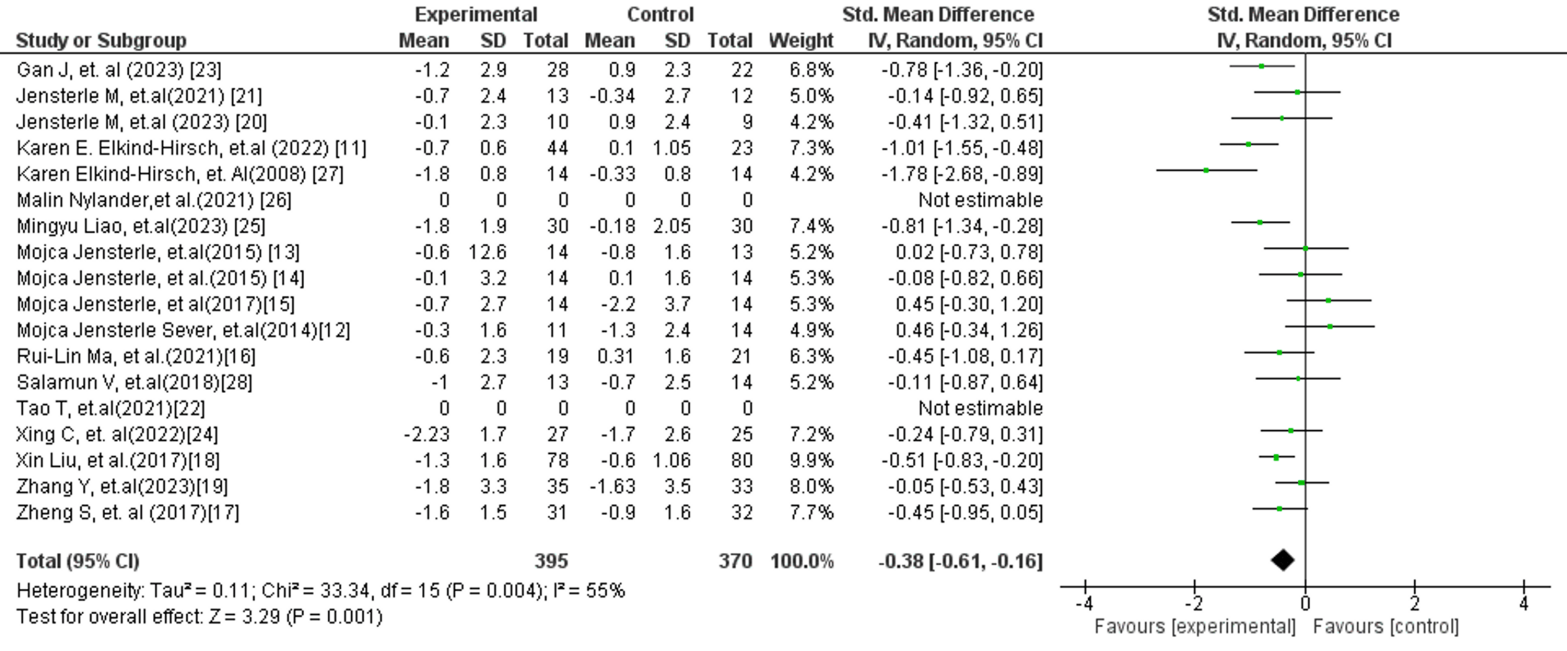
Forest plot for HOMA-IR overall HOMA-IR: Homeostatic model assessment of insulin resistance

In Figure 8, the overall pooled analysis demonstrated a statistically significant reduction in insulin resistance (HOMA-IR) favouring GLP-1 receptor agonists compared with control interventions (SMD: −0.38; 95% CI: −0.61 to −0.16; p = 0.001). Moderate heterogeneity was observed (I² = 55%), suggesting variability across studies, likely due to differences in study populations, intervention regimens, and treatment duration. Despite this, the direction of effect consistently favoured GLP-1 RA therapy, indicating a clinically meaningful improvement in insulin sensitivity.

**Figure 9:**
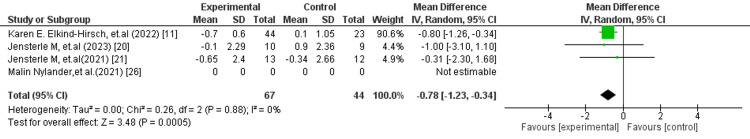
Forest plot comparing GLP-1 RA versus placebo for HOMA-IR GLP-1 RA: Glucagon-like peptide-1 receptor agonist; HOMA-IR: Homeostatic model assessment of insulin resistance

**Figure 10:**
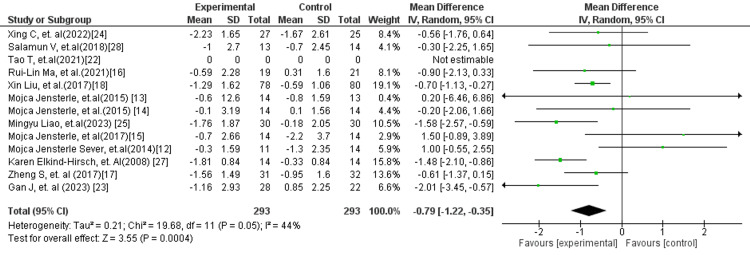
Forest plot comparing GLP-1 RA versus standard treatment for HOMA-IR GLP-1 RA: Glucagon-like peptide-1 receptor agonist; HOMA-IR: Homeostatic model assessment of insulin resistance

Among subgroup analyses, the comparison of GLP-1 receptor agonists versus placebo (Figure 9) showed a significant reduction in HOMA-IR (MD: −0.78; 95% CI: −1.23 to −0.34; p = 0.0005), with no heterogeneity (I² = 0%), indicating a consistent and reliable treatment effect. Similarly, when GLP-1 receptor agonists were compared with standard treatment (Figure 10), a significant improvement in insulin resistance was observed (MD: −0.79; 95% CI: −1.22 to −0.35; p = 0.0004), with moderate heterogeneity (I² = 44%). This supports the superiority of GLP-1 RAs over conventional therapies in improving metabolic parameters.

**Figure 11:**
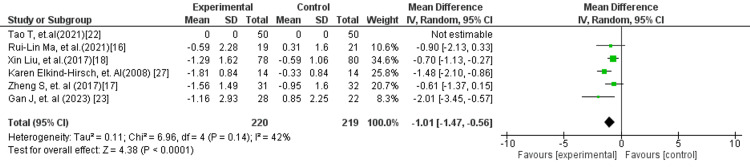
Forest plot comparing exenatide versus placebo/standard treatment for HOMA-IR HOMA-IR: Homeostatic model assessment of insulin resistance

Notably, the subgroup analysis of exenatide versus placebo/standard treatment (Figure 11) demonstrated a highly significant reduction in HOMA-IR (MD: −1.01; 95% CI: −1.47 to −0.56; p < 0.0001), with moderate heterogeneity (I² = 42%). The magnitude of effect in this subgroup was greater than that observed in the overall analysis, suggesting a particularly strong benefit of exenatide in improving insulin resistance.

**Figure 12:**
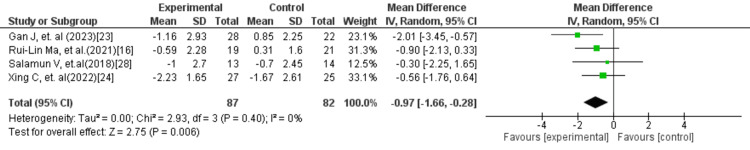
Forest plot comparing GLP-1 RA combined with metformin versus placebo/standard treatment for HOMA-IR GLP-1 RA: Glucagon-like peptide-1 receptor agonist; HOMA-IR: Homeostatic model assessment of insulin resistance

**Figure 13:**
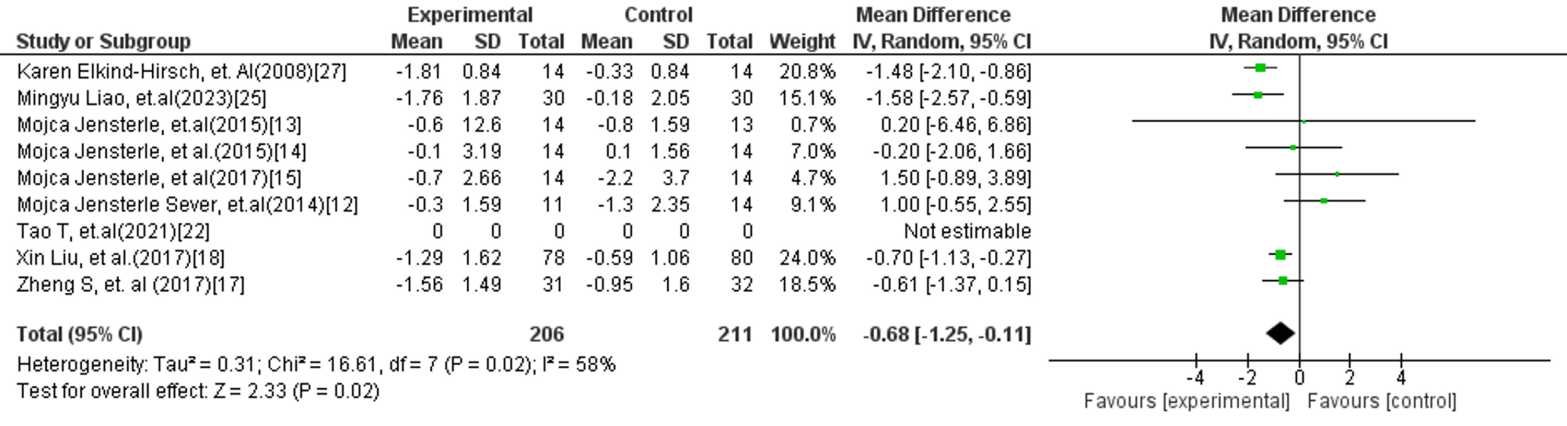
Forest plot comparing GLP-1 RA versus GLP-1 RA combined with metformin for HOMA-IR GLP-1 RA: Glucagon-like peptide-1 receptor agonist; HOMA-IR: Homeostatic model assessment of insulin resistance

In Figure 12, four studies including 169 participants were analysed to compare combination therapy with placebo or standard care. The pooled results showed that GLP-1 RA in combination with metformin significantly reduced HOMA-IR (MD = −0.97; 95% CI: −1.66 to −0.28; p = 0.006). No heterogeneity was detected (I² = 0%), suggesting consistent outcomes across studies. In Figure 13, eight studies with a total of 417 participants compared GLP-1 monotherapy to combination therapy. The analysis revealed that GLP-1 alone was associated with a modest but statistically significant reduction in HOMA-IR (MD = −0.68; 95% CI: −1.25 to −0.11; p = 0.02). However, moderate heterogeneity was observed (I² = 58%), indicating variability among study results.

**Figure 14:**
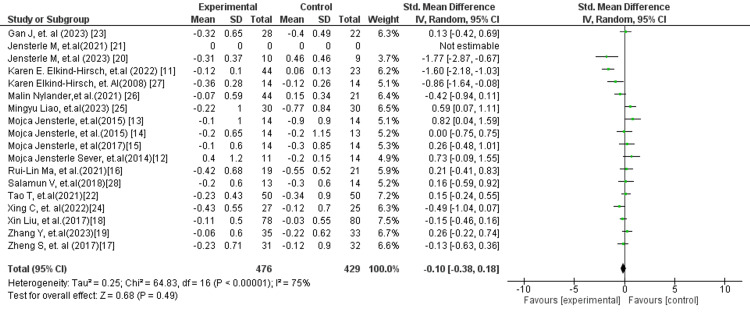
Forest plot for TT overall TT: Total testosterone

**Figure 15:**
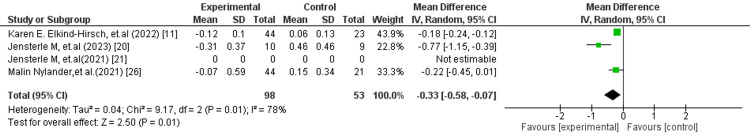
Forest plot comparing GLP-1 RA versus placebo for TT GLP-1 RA: Glucagon-like peptide-1 receptor agonist; TT: Total testosterone

In Figure 14, the overall pooled analysis demonstrated no statistically significant effect of GLP-1 receptor agonists on TT levels compared with control interventions (SMD: −0.10; 95% CI: −0.38 to 0.18; p = 0.49). Substantial heterogeneity was observed (I² = 75%), indicating considerable variability across the included studies. The CI crossing the line of no effect suggests that GLP-1 RAs do not consistently influence androgen levels in women with PCOS. However, subgroup analysis revealed a different pattern in specific comparisons. The GLP-1 receptor agonists versus placebo subgroup (Figure 15) demonstrated a statistically significant reduction in TT levels (MD: −0.33; 95% CI: −0.58 to −0.07; p = 0.01), although high heterogeneity was present (I² = 78%). This indicates that while GLP-1 RAs may exert a modest androgen-lowering effect in placebo-controlled settings, the results are inconsistent across studies.

Quality of the Included Studies

**Figure 16:**
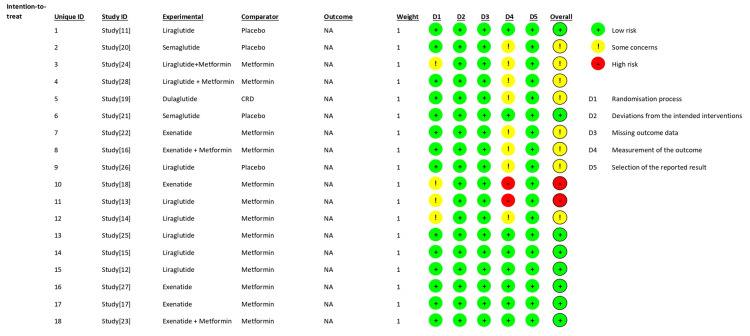
Figure depicting the risk of bias assessment of the included studies using RoB2

The overall risk of bias across the included studies was predominantly low, with most domains rated as low risk, as shown in Figure 16. However, some studies exhibited 'some concerns', particularly in domains related to deviations from intended interventions and outcome measurement. One study was judged to have a high risk of bias, primarily due to issues with missing outcome data. Overall, the methodological quality of the included trials was acceptable, although minor concerns in select domains should be considered when interpreting the pooled estimates.

**Figure 17:**
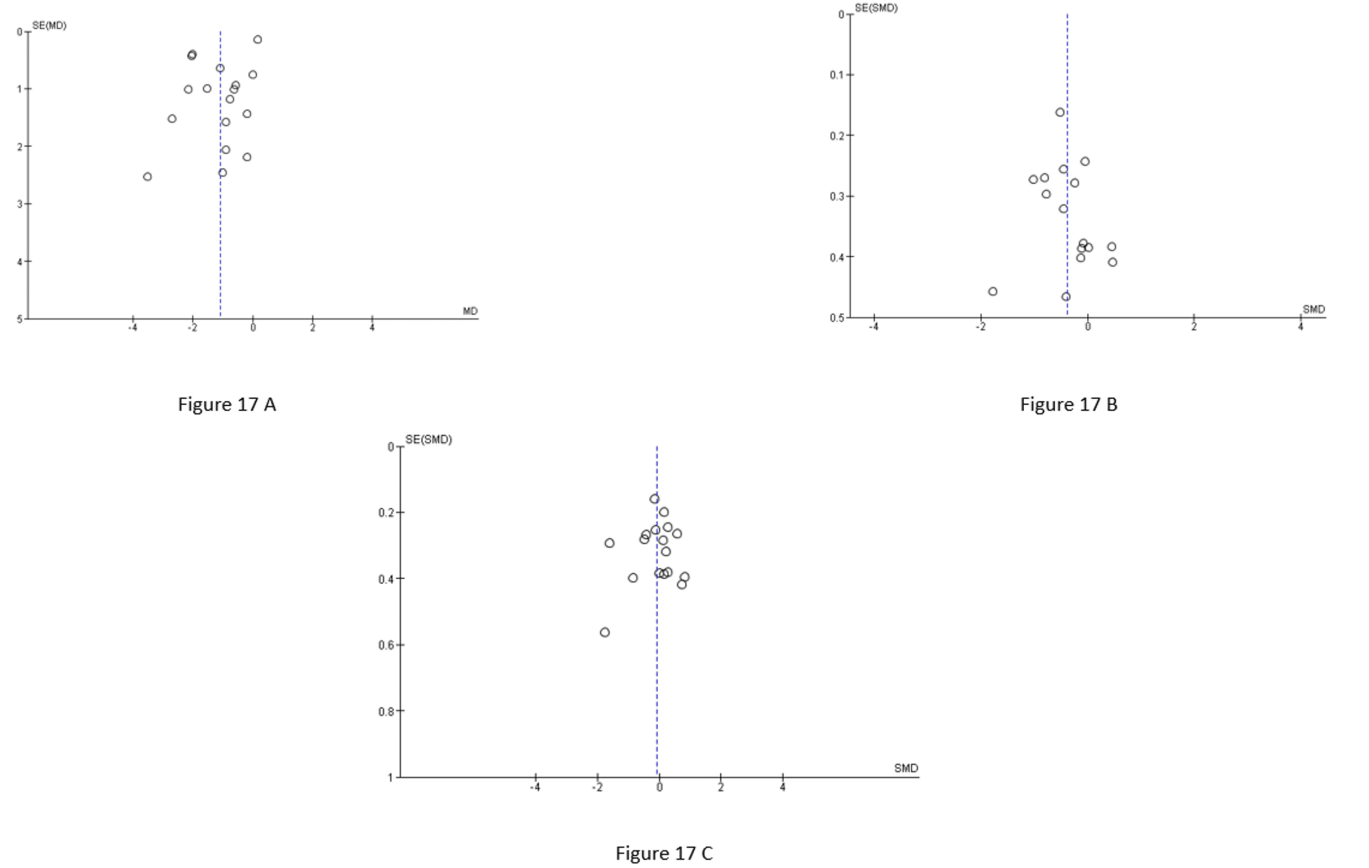
A, B, and C represent the funnel plots of the studies evaluated for BMI overall, HOMA-IR overall, and TT overall, respectively, generated using RevMan 5.4.1 HOMA-IR: Homeostatic model assessment of insulin resistance; TT: Total testosterone

The funnel plots for BMI (MD) (Figure 17A), HOMA-IR (SMD) (Figure 17B), and TT (SMD) (Figure 17C) appear largely symmetrical around the pooled effect estimate, with most studies distributed evenly on both sides of the vertical line. This suggests a low likelihood of significant publication bias. Overall, there is no strong visual evidence of substantial publication bias across the included outcomes.

GRADE Approach

**Figure 18:**
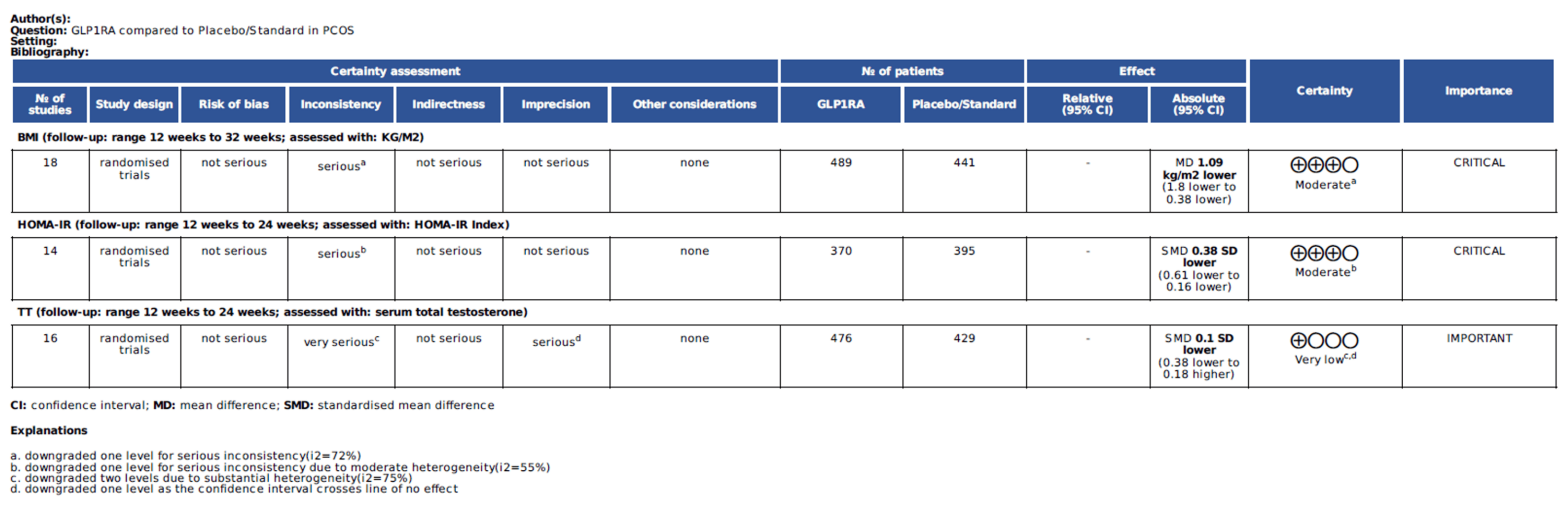
GLP-1 RA compared to placebo/standard for BMI, HOMA-IR, and TT certainty of evidence GLP-1 RA: Glucagon-like peptide-1 receptor agonist; HOMA-IR: Homeostatic model assessment of insulin resistance; TT: Total testosterone

The certainty of the evidence was determined using the GRADE approach (Figure 18). The certainty of evidence was moderate for BMI and HOMA-IR and very low for TT.

Discussion

In this study, the pooled findings demonstrate consistent and clinically meaningful improvements across all three outcomes of BMI, HOMA-IR, and TT, establishing GLP-1RAs as an effective pharmacological strategy in overweight and obese women with PCOS, either as monotherapy or combined with metformin [11].

BMI reduction was the most consistently reported outcome across the included trials, reflecting the potent weight-lowering properties of GLP-1RAs through central appetite suppression, delayed gastric emptying, and enhanced satiety signalling. In a phase 3 placebo-controlled trial, Elkind-Hirsch et al. demonstrated that liraglutide 3 mg produced significant and sustained BMI reductions in obese PCOS women over 32 weeks [11]. Jensterle Sever et al. confirmed that liraglutide combined with metformin achieved significant BMI reductions even in women previously unresponsive to metformin monotherapy [12]. Jensterle et al. reported that liraglutide monotherapy was superior to metformin for BMI reduction in a pilot trial [13], with a 12-week course producing clinically meaningful reductions in newly diagnosed PCOS [14]. Low-dose liraglutide combined with metformin achieved BMI reductions comparable to high-dose liraglutide alone, with implications for tolerability and cost [15]. Among exenatide trials, Ma et al. reported that combined exenatide and metformin produced greater BMI reductions than metformin alone [16], and Zheng et al. confirmed that exenatide monotherapy outperformed metformin for BMI reduction at 12 weeks [17]. Liu et al. and Frøssing et al. similarly observed significant BMI reductions with exenatide and liraglutide, respectively [18]. Zhang et al. demonstrated that dulaglutide combined with dietary restriction produced greater BMI reductions than diet alone [19], and semaglutide achieved BMI reductions consistent with its recognised superior weight loss profile in both the gastric emptying trial and the tongue fat pilot study [20,21].

Insulin resistance, indexed by HOMA-IR, is a central pathophysiological driver of PCOS, sustaining compensatory hyperinsulinaemia that stimulates ovarian androgen synthesis and impairs folliculogenesis. GLP-1RAs reduce HOMA-IR through glucose-dependent insulin secretion, glucagon suppression, and weight loss-mediated improvement in peripheral insulin sensitivity. Tao et al. demonstrated that exenatide alone and combined with metformin significantly reduced HOMA-IR in PCOS women with prediabetes, with the combination arm yielding the greatest improvement [22]. Gan et al. provided molecular support for these findings, demonstrating that combination therapy enhanced AMP-activated protein kinase signalling and suppressed pro-inflammatory cytokines to a greater degree than metformin monotherapy, mechanistically explaining the superior insulin sensitisation observed [23]. For liraglutide-based regimens, Xing et al. confirmed that metformin plus liraglutide was superior to metformin alone in reducing HOMA-IR, with the addition of liraglutide conferring incremental insulin-sensitising benefits [24]. Liao et al. further corroborated significant HOMA-IR reductions with GLP-1RA-based treatment in overweight PCOS women, reporting improvements comparable to those achieved with hormonal contraceptive therapy for this parameter [25]. Collectively, these findings indicate that GLP-1RAs address insulin resistance through complementary mechanisms that are both weight-dependent and weight-independent, reinforcing their utility in PCOS management beyond simple adiposity reduction.

TT is the principal biochemical marker of hyperandrogenism in PCOS, directly underpinning hirsutism, acne, and menstrual irregularity. Nylander et al. provided direct evidence of this effect, reporting significant reductions in TT and the free androgen index with liraglutide, alongside improvements in the LH to FSH ratio and menstrual regularity [26]. Elkind-Hirsch et al. demonstrated that combined exenatide and metformin improved hormonal parameters, including TT and menstrual cyclicity, more effectively than either agent alone, supporting the additive anti-androgenic effects of combination therapy [27]. In this study, the pooled findings demonstrate clinically meaningful improvements in BMI and HOMA-IR, supporting GLP-1 RAs as a promising pharmacological strategy in overweight and obese women with PCOS; however, the evidence for improvement in TT was not statistically significant overall and was of very low certainty.

Several limitations of the included studies must be acknowledged. Individual trial sample sizes were generally small, with several studies characterised as pilot investigations, limiting the precision of pooled effect estimates. Follow-up duration was short in most trials, typically ranging from 12 to 32 weeks, which precludes conclusions about the durability of improvements in BMI, HOMA-IR, and TT. Heterogeneity in PCOS diagnostic criteria, inconsistent outcome reporting, and the predominant enrolment of overweight or obese women restrict generalisability to lean PCOS phenotypes. The concentration of studies in Chinese and European populations further limits external validity across ethnically diverse settings. Larger multicentre trials with standardised outcome reporting are warranted, as illustrated by the promising preliminary reproductive data reported by Salamun et al. [28]. The present review does not address the comparative efficacy of individual GLP-1 receptor agonists, and it remains unclear whether the observed therapeutic benefits represent a true class effect or are molecule-specific. Furthermore, a discussion of investigational agents currently in the pipeline was beyond the scope of the included studies and the prespecified objectives of this review. These represent important areas warranting dedicated future investigation.

## Conclusions

GLP-1 RAs are effective in improving metabolic outcomes in women with PCOS, particularly in reducing BMI and improving insulin resistance in the relatively short-term follow-up duration. However, their effect on hyperandrogenism, as measured by TT, remains inconclusive and has very low certainty. Overall, GLP-1 RAs represent a valuable therapeutic option, especially in overweight or obese individuals with PCOS, although further large-scale, long-term trials are required to establish their role in reproductive outcomes and long-term disease modification.

## Appendices

Search strategies for the following databases

PubMed: ("polycystic ovarian syndrome" OR pcos) AND ("glp-1 agonist" OR Exenatide OR Lixisenatide OR Liraglutide OR Semaglutide OR Dulaglutide OR Tirzepatide) "Filters: Randomized Controlled Trial"

Cochrane: ("polycystic ovarian syndrome" OR pcos) AND ("glp-1 agonist" OR Exenatide OR Lixisenatide OR Liraglutide OR Semaglutide OR Dulaglutide OR Tirzepatide) "Filters: Randomized Controlled Trial"

MEDLINE: ("polycystic ovarian syndrome" OR pcos) AND ("glp-1 agonist" OR Exenatide OR Lixisenatide OR Liraglutide OR Semaglutide OR Dulaglutide OR Tirzepatide) "Filters: Randomized Controlled Trial"

Embase: ('polycystic ovarian syndrome' OR pcos) AND ('glp-1 agonist' OR Exenatide OR Lixisenatide OR Liraglutide OR Semaglutide OR Dulaglutide OR Tirzepatide) 'Filters: Randomized Controlled Trial'

CINAHL: ("polycystic ovarian syndrome" OR pcos) AND ("glp-1 agonist" OR Exenatide OR Lixisenatide OR Liraglutide OR Semaglutide OR Dulaglutide OR Tirzepatide) "Filters: Randomized Controlled Trial"

Web of Science: ("polycystic ovarian syndrome" OR pcos) AND ("glp-1 agonist" OR Exenatide OR Lixisenatide OR Liraglutide OR Semaglutide OR Dulaglutide OR Tirzepatide) "Filters: Randomized Controlled Trial"

Scopus: ("polycystic ovarian syndrome" OR pcos) AND ("glp-1 agonist" OR Exenatide OR Lixisenatide OR Liraglutide OR Semaglutide OR Dulaglutide OR Tirzepatide) "Filters: Randomized Controlled Trial"
